# Agave-Laurate-Bioconjugated Fructans Decrease Hyperinsulinemia and Insulin Resistance, Whilst Increasing IL-10 in Rats with Metabolic Syndrome Induced by a High-Fat Diet

**DOI:** 10.3390/ph17081036

**Published:** 2024-08-06

**Authors:** Angélica Sofía González-Garibay, Georgina Sandoval, Omar Ricardo Torres-González, Blanca Estela Bastidas-Ramírez, Iván Moisés Sánchez-Hernández, Eduardo Padilla-Camberos

**Affiliations:** 1 Medical and Pharmaceutical Biotechnology Unit, Center for Research and Assistance in Technology and Design of the State of Jalisco, A.C. (CIATEJ), Av. Normalistas No. 800 Col. Colinas de la Normal, Guadalajara C.P. 44270, Jalisco, Mexico; 2 Department of Molecular Biology and Genomics, Institute of Research on Chronic Degenerative Diseases, University Center of Health Sciences, Universidad de Guadalajara, Sierra Mojada No. 950 Col. Independencia, Guadalajara C.P. 44340, Jalisco, Mexico

**Keywords:** adipokines, inflammation, adipose tissue, fructooligosaccharides, acylation

## Abstract

Metabolic syndrome (MetS) comprises a cluster of metabolic risk factors, which include obesity, hypertriglyceridemia, high blood pressure, and insulin resistance. The purpose of this study was to evaluate the effects of laurate-bioconjugated fructans on pro- and anti-inflammatory cytokines in Wistar rats with MetS induced by a high-fat diet. Laurate-bioconjugated fructans were synthesized with agave fructans, immobilized lipase B, and vinyl laureate as the acylant. Groups were fed a standard diet (NORMAL), a high-fat diet (HFD), or a high-fat diet plus laurate-bioconjugated fructans (FL PREV) for 9 weeks. A fourth group received a high-fat diet for 6 weeks, followed by simultaneous exposure to a high-fat diet and laurate-bioconjugated fructans for 3 additional weeks (FL REV). The dose of laurate-bioconjugated fructans was 130 mg/kg. Laurate-bioconjugated fructans reduced food and energy intake, body weight, body mass index, abdominal circumference, adipose tissue, adipocyte area, serum triglycerides, insulin, insulin resistance, and C-reactive protein but they increased IL-10 protein serum levels and mRNA expression. The impact of laurate-bioconjugated fructans on zoometric and metabolic parameters supports their potential as therapeutic agents to improve obesity, obesity comorbidities, insulin resistance, type 2 diabetes mellitus, and MetS.

## 1. Introduction

The risk factors associated with type 2 diabetes mellitus have been observed since the last century, and the term metabolic syndrome (MetS) has subsequently been coined. MetS comprises a cluster of metabolic risk factors, which include central obesity, impaired glucose metabolism, hypertriglyceridemia, high blood pressure, and low high-density lipoprotein cholesterol levels [1,2]. MetS is diagnosed when at least three of the above criteria are met; if possible, insulin resistance should also be considered [3]. An accumulation of the risk factors increases the likelihood of developing type 2 diabetes mellitus, cardiovascular disease, and strokes [4].

Obesity is strongly related to inflammation. This is because of the accumulation of adipose tissue primarily characterized by adipocyte hypertrophy. Consequently, the expression of adipose tissue adipokines—including interleukin 6 (IL-6), tumor necrosis factor-alpha (TNF-α), adiponectin [5,6,7], interleukin 7 (IL-7) [6,8,9], and interleukin 10 (IL-10)—changes [6,10]. In addition, C-reactive protein (CRP) is an acute-phase reactant; it is considered to be a marker of low-grade inflammation in humans [11]. A negative correlation has been observed between adiponectin and proinflammatory cytokines such as IL-6, TNF-α, and CRP in humans [12,13].

Low-grade inflammation is related to insulin resistance (IR) [14]. Homeostasis model assessment of insulin resistance (HOMA-IR) has been widely used for the estimation of IR in humans and experimental animal models [15,16], and it is considered a valid measure to determine insulin resistance in Wistar rats [17]. Chronic hyperinsulinemia and insulin resistance eventually lead to hypertension, reduced insulin secretion, and hyperglycemia. Prolonged hyperglycemia ultimately results in the diagnosis of type 2 diabetes mellitus [18,19,20].

Obesity is associated with an increased risk of non-alcoholic fatty liver disease (NAFLD), which consists in the accumulation of hepatic triglycerides. NAFLD is a manifestation of MetS in the liver [21].

It is estimated that one-quarter of the global population suffers from MetS. In the United States, it is approximately one-third [22,23,24]. Patients diagnosed with MetS are advised to lose weight, exercise, and consume a healthy diet [13,25]. Many studies have demonstrated a positive association between saturated fatty acid (SFA) intake and MetS factors. Consequently, most dietary guidelines around the globe recommend limiting SFA [26,27,28].

In Mexico, agave plants are used in the production of alcoholic beverages. The leaves of these plants are crop residues that are rich in fibers called fructooligosaccharides or fructans [29,30]. Agave fructans are defined as polymers according to their terminal or internal glucose units and are linked by β (2 → 1) and β (2 → 6) bonds, as stated in the Mexican Standard NMX-F591-SCFI [31]. Agave fructans have been reported to exert beneficial effects on serum glucose concentrations, body weight, and the immune system by acting as prebiotics stimulating the growth of beneficial bacteria [32]. A previous study demonstrated that the ingestion of fructooligosaccharides in rats led to changes in adipokine secretion from mesenteric adipocytes. These effects were attributed to either the intact form of the fructooligosaccharides or their fermentation products [33]. Fermentation products promote the production of peptides involved in appetite regulation, which positively affects glucose metabolism, body weight, fat mass, and associated metabolic disorders [34,35]. On the other hand, laurate, or lauric acid (C12:0), is a medium-chain saturated fatty acid known for its cardiometabolic benefits. It is commonly used as a weight-loss supplement or energy source due to its ability to be rapidly oxidized without requiring carnitine transport into mitochondria and therefore without being stored in the body [36,37].

Fructans from *Agave tequilana* Weber var. *azul* have been functionalized using organic [38] or enzymatic acylation with fatty acids [39,40] to generate laurate-bioconjugated fructans. Recent studies have demonstrated that laurate-bioconjugated fructans improve glucose and lipid metabolism, but their impact on zoometric parameters has differed in rats with MetS [40,41].

Different diets induce varying outcomes in the characteristics of MetS [5,42]. In the present study, we utilized a high-fat diet (mainly lard) because it has been demonstrated to lead to metabolic disturbances such as obesity, hyperglycemia, and hypertriglyceridemia in Wistar rats [43,44,45].

In this study, we evaluated the prevention and reversion effects of laurate-bioconjugated fructans on obesity, insulin resistance, blood pressure, and pro- and anti-inflammatory adipokines (protein serum levels and mRNA) in Wistar rats with MetS induced by a high-fat diet.

## 2. Results

### 2.1. Laurate-Bioconjugated Fructan Synthesis

**Figure 1:**
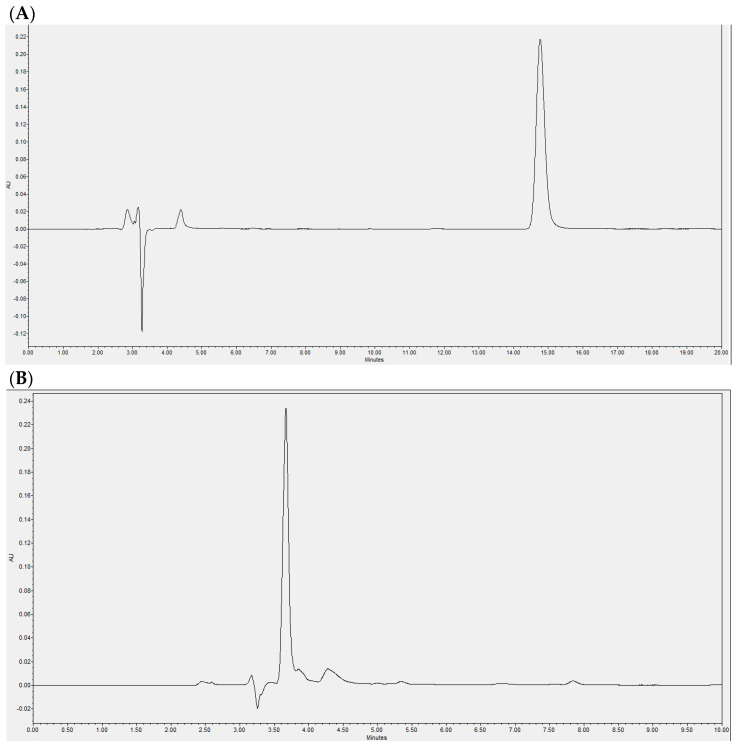

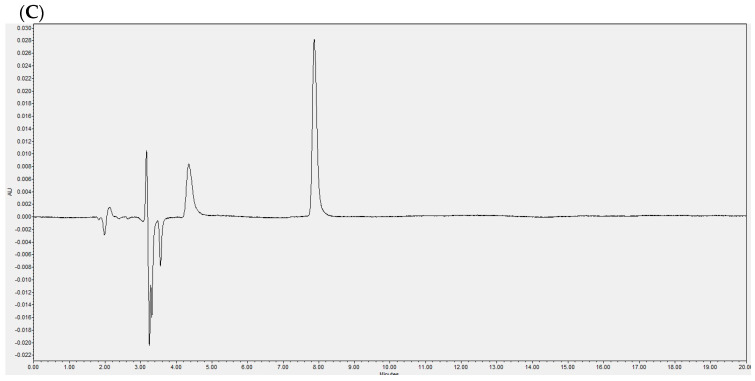
Representative chromatograms by HPLC-DAD separation. (**A**) Chromatogram showing the vinyl laureate peak in the sample at the beginning of the reaction; (**B**) chromatogram of the product sample of the enzymatic reaction after 48 h; (**C**) typical chromatogram of lauric acid.

The enzymatic reaction was evaluated at the beginning using HPLC-DAD, revealing a single peak corresponding to vinyl laureate (Figure 1A). The product profile of the enzymatic reaction was determined at the end of 48 h, showing a main peak evidencing the presence of laurate-bioconjugated fructans and the complete consumption of vinyl laurate (Figure 1B). A typical chromatogram of lauric acid is shown in Figure 1C.

### 2.2. Laurate-Bioconjugated Fructans Reduce Food and Energy Intake

The animals treated with laurate-bioconjugated fructans during the entire intervention significantly (*p* < 0.0001) reduced their amount of food intake compared with the high-fat-fed animals (FL PREV vs. HFD: 14.8 ± 2.5 g/day vs. 18.4 ± 2.1 g/day, respectively). This effect also occurred in animals treated with laurate-bioconjugated fructans for the final 3 weeks (FL REV vs. HFD: 16.5 ± 3.6 g/day vs. 18.4 ± 2.1 g/day, respectively). The energy intake was also lower when compared with that of the animals treated with laurate-bioconjugated fructans for 9 weeks (FL PREV vs. HFD: 68.2 ± 11.6 kcal/day vs. 84.7 ± 9.5 kcal/day, respectively) and also when animals were treated for the final 3 weeks (FL REV vs. HFD: 76.0 ± 16.7 kcal/day vs. 84.7 ± 9.5 kcal/day, respectively). There were no differences in energy intake between the HFD group and the NORMAL group (Table 1).

The daily energy intake from fat was lower in the NORMAL group because of the intrinsic differences in the amount of fat in the high-fat diet compared with the standard diet. The FL PREV group consumed less energy (*p* < 0.0001) from fat than the high-fat group (31.2 ± 5.3 kcal/day vs. 38.7 ± 4.3 kcal/day, respectively). Similarly, FL REV also consumed less fat than HFD (34.7 ± 7.6 vs. 38.7 ± 4.3 kcal/day, respectively).

### 2.3. Laurate-Bioconjugated Fructans Prevent Body Weight Gain, Reverse Obesity, and Improve Metabolic Parameters

Compared with the group receiving high-fat diet, FL PREV reduced the abdominal circumference of the rats (26.4 ± 0.5 cm vs. 27.2 ± 0.4 cm, respectively). The fasting serum glucose levels at 9 weeks were not significantly different for the FL PREV group compared with HFD (134.3 ± 14.6 vs. 148.3 ± 12.4 mg/dL, respectively), nor in the FL REV group vs. HFD (125.8 ± 22.2 vs. 148.3 ± 12.4 mg/dL, respectively) (Table 2). Although the serum triglycerides levels were not significantly different, the groups treated with laurate-bioconjugated fructans had slightly lower mean levels (FL PREV 66.7 ± 24.9 mg/dL vs. HFD 99.1 ± 15.2 mg/dL, *p* = 0.08; FL REV 70.7 ± 10.6 mg/dL vs. HFD 99.1 ± 17.0 mg/dL, *p* = 0.09). Total cholesterol did not differ between the groups.

The adiponectin, TNF-α, and IL-6 levels were not significantly different between the HFD and NORMAL groups or between the groups treated with laurate-bioconjugated fructans and the HFD group.

**Figure 2:**
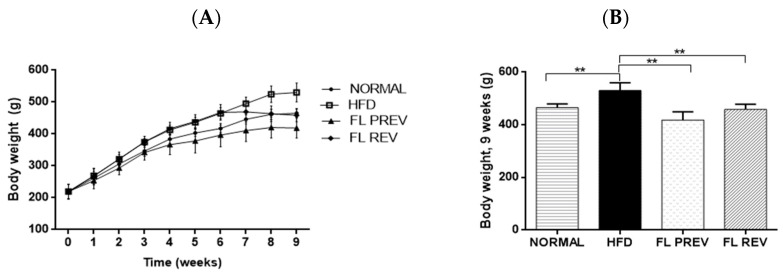
Effect of laurate-bioconjugated fructans on the body weight of rats fed a high-fat diet. (**A**) Trends in body weight during the entire intervention. (**B**) Final body weight after 9 weeks. NORMAL: rats fed a standard diet for 9 weeks. HFD: high-fat diet. FL PREV: high-fat diet for 9 weeks plus laurate-bioconjugated fructans. FL REV: high-fat diet for 6 weeks then high-fat diet plus laurate-bioconjugated fructans for 3 additional weeks. ** *p* < 0.01.

The intervention with laurate-bioconjugated fructans for 9 weeks in the FL PREV group prevented body weight gain compared with the HFD group (418 ± 31 g vs. 530 ± 29 g, respectively; *p* < 0.01). The FL REV group, which consisted of animals who received a high-fat diet for 6 weeks and then laurate-bioconjugated fructans along with a high-fat diet for 3 weeks, lost significantly more body weight (*p* < 0.01) vs. the HFD group (458 ± 20 g vs. 530 ± 29 g, respectively) (Figure 2).

### 2.4. Laurate-Bioconjugated Fructans Prevent Adipose Tissue Accumulation with No Effect on Liver Histology and Weight

**Figure 3:**
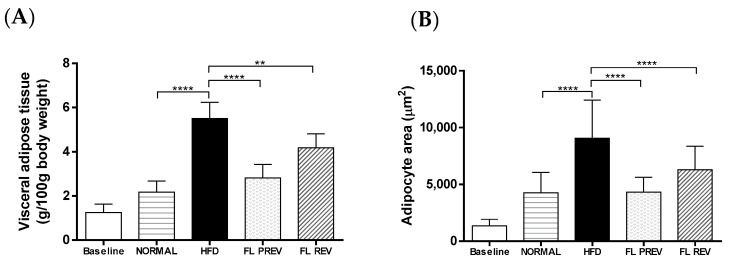

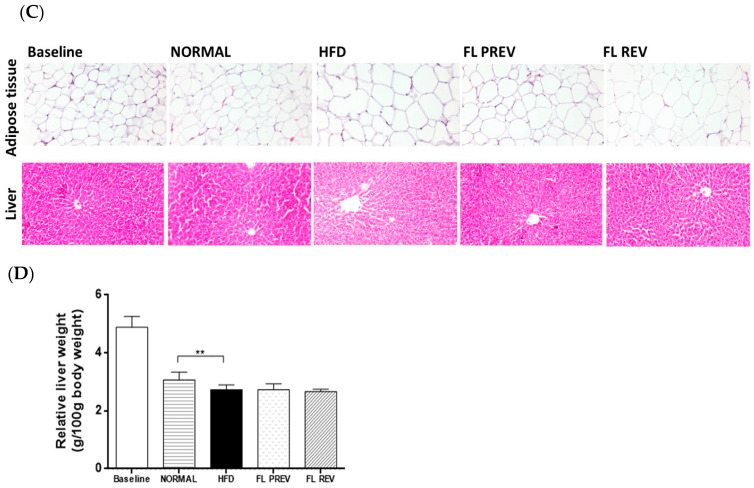
Effects of laurate-bioconjugated fructans on adipose and liver tissues in rats fed a high-fat diet. (**A**) Visceral adipose tissue relative to body weight. (**B**) Quantification of the adipocyte area in H&E-stained adipose tissue. (**C**) Adipose and liver histology of the treated animals. Representative photomicrographs after H&E staining, 20× magnification. (**D**) Relative ratio of fresh liver weight to body weight. NORMAL: animals fed a standard diet for 9 weeks. HFD: high-fat diet. FL PREV: high-fat diet for 9 weeks plus laurate-bioconjugated fructans. FL REV: high-fat diet for 6 weeks then high-fat diet plus laurate-bioconjugated fructans for 3 additional weeks. ** *p* < 0.01; **** *p* < 0.0001. The baseline group was not included in the statistical analysis.

Visceral adipose tissue was measured at the end of the study and normalized to 100 g of body weight. The FL PREV group accumulated significantly less adipose tissue compared with the HFD group (2.8 ± 0.6 g vs. 5.5 ± 0.7 g, respectively). Similarly, the FL REV group gained less adipose tissue vs. the HFD group (4.2 ± 0.6 g vs. 5.5 ± 0.7 g, respectively). Visceral adipose tissue was not significantly different between the FL PREV group and the NORMAL group (2.8 ± 0.6 g vs. 2.2 ± 0.5 g, respectively) (Figure 3). Liver weight was not significantly different between the groups receiving laurate-bioconjugated fructans and the HFD group, likewise histological features and NAS score were not different (Figure 3, Table 3).

### 2.5. Laurate-Bioconjugated Fructans Reduce Visceral Adipocyte Area

An analysis of the adipocyte size revealed that the treatment with laurate-bioconjugated fructans for 9 weeks significantly prevented an increase in the adipocyte area (Figure 3). The adipocyte area of the FL PREV group only reached 4321 ± 1298 μm^2^, whereas that of the HFD group reached 9064 ± 3356 μm^2^ (*p* < 0.0001). The reversion treatment with laurate-bioconjugated fructans significantly reduced the adipocyte size of the FL REV group (6284 ± 2073 μm^2^ vs. 9064 ± 3356 μm^2^ for the HFD group; *p* < 0.01). The high-fat diet significantly increased the adipocyte size of the HFD group compared with the NORMAL group fed with a standard diet (9064 ± 3356 μm^2^ vs. 4257 ± 1804 μm^2^, respectively). The adipocyte size of the FL PREV group was similar to that of the NORMAL group (4321 ± 1298 μm^2^ vs. 4257 ± 1804 μm^2^; not significant).

### 2.6. Laurate-Bioconjugated Fructans Reduce Systolic Blood Pressure and Insulin Resistance

The group that received laurate-bioconjugated fructans for 9 weeks (FL PREV) presented a decrease in systolic blood pressure compared with the HFD group (110.7 ± 14.1 mm Hg vs. 135.8 ± 6.3 mm Hg, respectively). This was also observed in the group that was administered laurate-bioconjugated fructans for the last 3 weeks of the study (FL REV) vs. the HFD group (127.2 ± 9.9 mm Hg vs. 135.8 ± 6.3 mm Hg, respectively).

**Figure 4:**
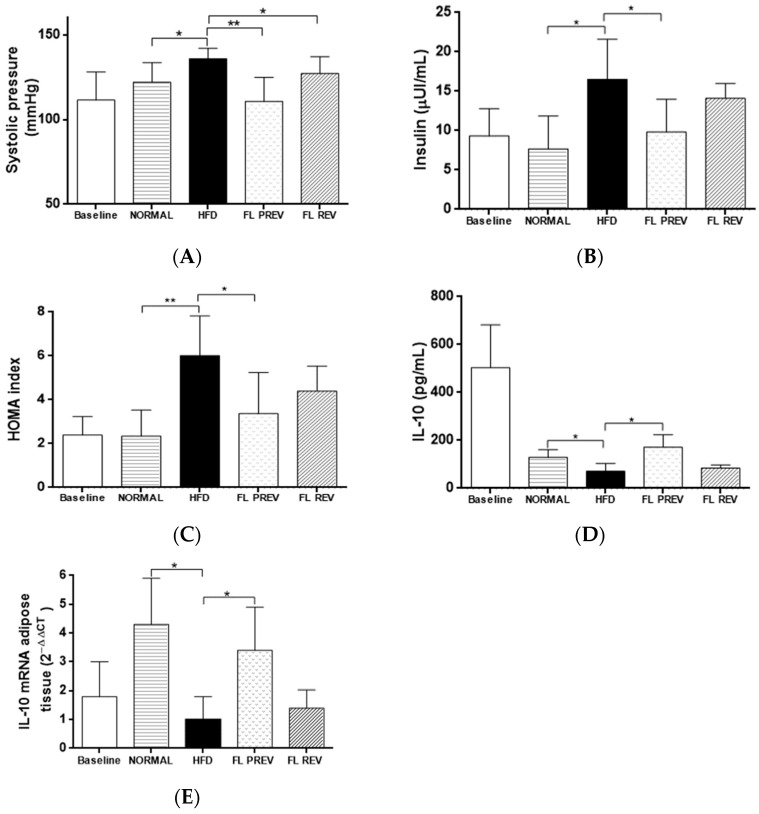
Effect of laurate-bioconjugated fructans on insulin, HOMA index, systolic pressure, and anti-inflammatory adipokine IL-10. (**A**) Systolic blood pressure. (**B**) Fasting insulin. (**C**) Homeostasis model assessment of insulin resistance (HOMA index). (**D**) IL-10 serum protein levels. (**E**) mRNA expression of visceral adipose tissue IL-10 analyzed using the comparative relative expression CT (2^−ΔΔCT^). NORMAL: standard diet for 9 weeks. HFD: high-fat diet. FL PREV: high-fat diet for 9 weeks plus laurate-bioconjugated fructans. FL REV: high-fat diet for 6 weeks then high-fat diet plus laurate-bioconjugated fructans for 3 additional weeks. * *p* < 0.05, ** *p* < 0.01. The baseline group was not included in the statistical analysis.

Hyperinsulinemia was present in the HFD group at the end of the study compared with the NORMAL group (16.5 ± 5.1 μUI/mL vs. 7.7 ± 4.2 μUI/mL, respectively). The FL PREV group had lower insulin levels than the HFD group (9.8 ± 4.1 μUI/mL vs. 16.5 ± 5.1 μUI/mL, respectively) (Figure 4). In accordance with these results, the HOMA index was higher in the HFD group than in the NORMAL group (6.0 ± 1.6 vs. 2.4 ± 1.1, respectively). Treatment with laurate-bioconjugated fructans for 9 weeks significantly decreased the HOMA index in the FL PREV group compared with the HFD group (3.4 ± 1.7 vs. 6.0 ± 1.6, respectively). The FL REV group exhibited an improvement in the HOMA index, but it was not significant vs. the HFD group (4.2 ± 0.8 vs. 6.0 ± 1.6, respectively).

### 2.7. Effects of Laurate-Bioconjugated Fructans on Pro- and Anti-Inflammatory Markers

The TNF-α, adiponectin, and IL-7 serum levels were not significantly different among the groups at the end of the study. IL-6 was slightly lower in the FL PREV group compared with the HFD group (3.3 ± 0.7 ng/mL vs. 4.7 ± 1.7 ng/mL, respectively) but the difference was not statistically significant. The CRP levels were significantly lower in the FL PREV group than in the HFD group (266.0 ± 49.5 μg/mL vs. 335.6 ± 24.2 μg/mL, respectively). The HFD group had lower levels of the anti-inflammatory cytokine IL-10 than the NORMAL control group at the end of the intervention (71.1 ± 32.5 pg/mL vs. 128.9 ±32.5 pg/mL, respectively). Our study revealed that laurate-bioconjugated fructans increased the IL-10 level in the FL PREV group vs. the HFD group (171.9 ± 51.9 pg/mL and 71.1 ± 32.5 pg/mL, respectively).Laurate-bioconjugated fructans also increased the mRNA expression of IL-10 in adipose tissue 3.4-fold (Figure 4). We did not observe significant differences in the patterns of TNF-α, adiponectin, IL-6, or IL-7 mRNA expression evaluated in the adipose tissue.

## 3. Discussion

In this study, we examined—for the first time—the reversal effect of laurate-bioconjugated fructans on the parameters of MetS and their preventative effects in rats with MetS induced by a high-fat diet. The rats fed a high-fat diet developed MetS; this was ascertained from the differences observed in body weight, adipose tissue, adipocyte size, systolic blood pressure, and insulin resistance compared with rats fed a standard diet.

The emergence of MetS occurred despite the comparable energy intake between the HFD and NORMAL groups. This finding correlated with previous reports [5,46]. Although we previously utilized a model with a hypercaloric diet (high in fructose) combined with laurate-bioconjugated fructans, this time, we report again triglycerides, cholesterol, glucose levels and blood pressure because Francisqueti et al. emphasized the significance of dietary composition, particularly the fat profile, in the development of MetS. Regarding serum glucose, our previous work showed that laurate-bioconjugated fructans significantly decreased serum glucose levels, whereas in this study, the reduction did not reach statistical significance. The observed benefits on glucose levels may not be directly attributed to glucose absorption itself, but rather to the effects of fructan fermentation [34,35,47]. In this study, serum triglycerides, blood pressure, and adipose tissue increased in rats fed a high-fat diet. In our previous work and in the present study, intervention with laurate-bioconjugated fructans reduced blood pressure, while triglycerides showed a slight decrease in the present study.

The impact of laurate-bioconjugated fructans on blood pressure was investigated by Hernández-Pérez et al. through the evaluation of antioxidant enzymes, specifically catalase and superoxide dismutase, in the hearts of rats subjected to a high-fat high-carbohydrate diet. However, they did not observe any significant differences [41]. In the current study, we observed a reduction in blood pressure accompanied by a decrease in hyperinsulinemia and insulin resistance. Insulin resistance and the resulting compensatory increase in plasma insulin levels activate mechanisms that enhance renal sodium reabsorption and stimulate sympathetic nervous system activity. These physiological changes can elevate blood pressure and potentially lead to hypertension [20].

Visceral adipose tissue depots (mesenteric, omental, perigonadal, perirenal, and retroperitoneal) are similar in humans and rodents. One distinction is that rodents have large perigonadal/epididymal adipose tissue, whereas humans have detectable omental adipose tissue [48,49]. Elevated levels of IL-6 and TNF-α proteins were observed in the epididymal adipose tissue of rats fed a high-fat diet beginning at week 24 when measured using Western blotting. This augmentation in adipokines was detected using direct measurements of the adipose tissue [5]. One study measured CRP, IL-6, and TNF-α serum levels, revealing elevated levels of IL-6 and TNF-α [50]. Another study reported higher serum IL-6, IL-10, and TNF-α in rats with MetS compared with a control group [7]. Our results deviated from those reported in prior research. It is crucial to acknowledge that this disparity could extend to humans as well. A recent study demonstrated that the serum levels of a large set of serum cytokines and adipokines, including those assessed in our investigation, exhibited no significant differences between patients with metabolically unhealthy obesity and those with metabolically healthy obesity. Metabolically healthy obesity is characterized by a metabolic profile similar to that of healthy non-obese individuals [51].

Energy intake was lower in groups treated with laureate-bioconjugated fructans compared to the high-fat diet-fed group. Some studies have shown that a specific type of fructan increases the levels of satiogenic peptides such as polypeptide YY and glucagon like peptide 1 and suppresses appetite in humans, although results are still conflicting [35,52,53]. Other benefits have been observed, such as a tendency to decrease ghrelin levels and affect energy intake and insulin levels, although these changes were not statistically significant [52]. It has been proposed that fermentation of fructans causes a differential release of peptides that regulate food intake [53]. Therefore, it is possible that laurate-bioconjugated fructans may alter the levels of gut peptides related to appetite, thereby affecting insulin levels and consequently influencing blood pressure.

Acylation causes important changes in the physicochemical properties of agave fructans; for instance, their water- and oil-holding capacities are higher [54]. These changes might impact food intake—and, therefore, body weight—in animals. The anti-obesity activity of laurate-bioconjugated fructans has previously been reported [40]. Our results revealed that laurate-bioconjugated fructans fully prevented gains in body weight and adipose tissue. They also reversed obesity, as evidenced by the body weight and visceral adipose tissue measurements. Laurate-bioconjugated fructans also prevented adipocyte hypertrophy. Our results for body weight were contrary to a previous study that revealed no effect on body weight or adipose tissue when laurate-bioconjugated fructans were used despite using the same Wistar strain [41]. This might be attributable to variables in the experimental design such as rat age. Hernández-Pérez et al. included 21-day-old rats; in our study, the rats were 8 weeks old. Another factor could be the number of locations of adipose tissue considered; we collected mesenteric, retroperitoneal, epididymal, and perirenal fat depots, whereas Hernández-Pérez et al. separately analyzed epididymal and abdominal fat tissue depots. Also, they provided a high-fat and high-carbohydrate diet (20% fat and 59% carbohydrate), whereas we fed the rats a high-fat diet consisting of 45.7% fat.

Our study provides further evidence of the effects of laurate-bioconjugated fructans on adipose tissue, not only because they prevented adipocyte hypertrophy (as evidenced by the adipocyte area), but also because they reversed the increase in the adipocyte size. Strikingly, the adipocyte area of animals fed a high-fat diet and laurate-bioconjugated fructans for 9 weeks was similar to that of animals fed a standard diet.

Supporting the benefits of fructans on adipose tissue, one prior study fed one group of rats with a diet rich in sucrose containing 5% fructans for 5 weeks and another group was fed with the same diet for 3 weeks plus 2 weeks of the same diet supplemented with fructans. Although no differences in body weight were observed, the fat pads from several adipose tissue depots were significantly lower in the animals treated with fructans. The mesenteric fat cell size was also measured and no difference was observed. The study included 3-week-old rats, acknowledging that the required age of rats to collect enough abdominal tissue is 8 weeks [48]. Therefore, the development of adipose tissue and the fat deposits evaluated must be considered when making comparisons. In our study, the rats were already 8 weeks of age at the baseline.

The effects of laurate-bioconjugated fructans on body weight and adipose tissue might be related to fructans per se. Fructan supplementation (15% from diet) from *Agave angustifolia* was demonstrated to decrease food intake, body weight, and metabolic parameters such as glucose, low-density lipoprotein cholesterol, and liver steatosis in rats fed a commercial rodent diet enriched with 10% coconut oil and 15% fructose [55].

The adiponectin serum levels did not change among the groups in our study. This finding concurred with an 18-week intervention using Wistar rats who received a high-fat diet. No difference was observed in the serum adiponectin levels when compared with rats fed a standard diet, although the adiponectin mRNA from the epididymal adipose tissue was lower compared with the control group [42]. Another study revealed that plasma adiponectin in rats fed 32.5% fat was not different from that of rats who received a standard diet for 10 weeks [56]. One prospective study of a rat model concluded that there was no association between MetS induced by diet and adiponectin levels after an intervention of 26 weeks. The adiponectin levels changed over time and were higher in the group fed a high-fat diet at 2, 18, and 26 weeks but similar to those of the control group at 48 weeks [57]. Another study also revealed an increase in the mean adiponectin levels in rats fed a high-fat diet, although changes in adiponectin levels were observed during the day [58]. Adiponectin levels seem to vary during the course of MetS and many authors have observed hypoadiponectinemia in humans and animals with obesity compared with non-obese individuals. It has also been observed that adiponectin levels further decrease in patients with coexisting type 2 diabetes mellitus. Other studies have not proved these changes [59].

A recent systematic review of the effects of fructooligosaccharides on inflammation and immunomodulation concluded that fructooligosaccharide supplementation is positively associated with an anti-inflammatory effect that may be beneficial for the health of the host. This study highlighted the effects on IL-6 and IL-10 (among other cytokines) in the gut [60]. Rendón-Huerta et al. [55] revealed that a 15% fructan supplementation for 24 weeks in obese diabetic rats fed a high-fat and high-fructose diet resulted in a diminution of TNF-α and IL-6 levels. In our study, laurate-bioconjugated fructans did not significantly change TNF-α or IL-6 but they slightly decreased the IL-6 concentration. Additionally, the high-fat diet did not significantly increase adipokines in the HFD group compared with the NORMAL group. However, Rendón-Huerta et al. only used fructans and their study was longer. This highlights the importance of the interplay within adipokines as well as the overall changes in their expression over time.

A negative correlation has been described between fasting insulin and CRP levels. CRP expression in the liver is predominantly controlled by IL-6 [61,62]. In our study, laurate-bioconjugated fructans significantly diminished the insulin levels and HOMA indices in the FL PREV group. Benefits were also observed in the FL REV group. These might be attributable to the finding of diminution in energy intake in both groups. The importance of investigations directed towards finding reversal options for metabolic diseases is crucial, such as a previous review and a meta-analysis where non-pharmacological interventions such as diet and certain pharmacological approaches reversed pre-diabetes and type 2 diabetes mellitus [63,64].

In our study, the CRP levels were lower in the FL PREV group, although no significant differences were observed between the HFD and NORMAL groups. Similarly, a recent study reported that a treatment with 10% fructooligosaccharide decreased CRP levels in rats, and fructooligosaccharides demonstrated superior efficacy in reducing CRP levels compared with mannooligosaccharides or galactooligosaccharides. In contrast to our findings, the aforementioned study revealed that a high-fat diet increased CRP levels compared with rats fed a standard diet [65]. In a randomized controlled clinical trial, women with type 2 diabetes mellitus received oligofructose-enriched inulin, a prebiotic similar to fructooligosaccharide. The trial presented a decrease in CRP levels [66]. Another study showed that fructans slightly lowered the IL-6 concentration. These effects might be related to their prebiotic effect because fructans increase beneficial gut bacteria such as bifidobacteria and augment short-chain fatty acids, reducing inflammation [60,61]. An increase in CRP serum levels increases the odds of the incidence and persistence of MetS, as revealed in a follow-up study that included male and female adult subjects [11].

An intervention study in which rats consumed a high-fat and fructose diet for 7 weeks revealed lower levels of IL-10, but the IL-10 levels increased when the rats were fed a high-fiber diet with up to 20.25% fiber [67]. In a clinical trial, 15 g fructooligosaccharides was administered to patients with Crohn’s disease. This supplementation increased the IL-10 expression in mucosal dendritic cells [68]. In another clinical trial, female participants diagnosed with type 2 diabetes mellitus were administered oligofructose-enriched inulin. The findings indicated a trend towards elevated levels of IL-10 but without statistical significance [66]. Similarly, laurate-bioconjugated fructans increased IL-10 serum protein and mRNA levels in the adipose tissue of the prevention group in our study but only a slight increase was observed in the reversion group. This improvement corroborated the findings of Zahedi et al. [11]; their follow-up study revealed that the serum IL-10 concentration slightly increased in their recovery MetS group compared with the MetS group.

The impact of laurate-bioconjugated fructans on systolic blood pressure observed in our study concurred with the benefits reported by Hernández-Pérez et al. This benefit might be related to the prebiotic effect of agave fructans, which increase the growth of bifidobacteria and lactobacilli. It has been demonstrated that obese hypertensive adult subjects have less bacterial diversity in the gastrointestinal tract than non-obese subjects [13,69]. Moreover, a decrease in mean blood pressure was noted with the acute administration of lauric acid (3–10 mg/kg), to Wistar and spontaneously hypertensive rats [36].

The benefits of laurate-bioconjugated fructans for insulin resistance observed in our study might also be related to lauric acid [70]. The effects of lauric acid on metabolic disorders in mice fed a high-fat diet have been explored and have revealed improvements in insulin and insulin resistance, for example, in one study, a high-fat diet containing 3% lauric acid was administered to mice [71]. In another study, lauric acid accounted for 1169 mg/kg of the diet, corresponding to 0.12% [37]. Although the total quantity of lauric acid differed from the amount administered in this study, the impact of lauric acid by itself on metabolic effects must be considered in future studies. Administering diets containing 42–59% of total energy from fat, derived from coconut oil or medium-chain triglyceride oil rich in lauric acid, to mice or rats for a duration of 5–12 weeks preserved insulin sensitivity and glucose tolerance comparable to those observed in animals fed a standard chow diet. This effect contrasts with the outcomes observed in diets rich in either saturated or unsaturated long-chain fatty acids [72,73]. In humans, small amounts of medium-chain fatty acids have been found to protect against insulin resistance in conditions of lipid-induced energy excess [74].

A recent study using Sprague Dawley rats with NAFLD who were fed a high-fat diet and concurrently administered lauric acid at doses of 250 and 500 mg/kg presented improvements similar to the findings in our study. These improvements included reductions in serum triglycerides, blood glucose, and insulin levels as well as an increase in IL-10 [75]. On the other hand, Shinoki et al. observed benefits to insulin and insulin resistance in rats fed a high-sucrose diet plus fructans [33].

## 4. Materials and Methods

### 4.1. Synthesis of Agave Fructan Bioconjugates

Agave fructan bioconjugates were synthesized in accordance with procedures described in previous works [39,40,41] and in patent MX 358,789 [76]. In this process, branched fructans were acylated using vinyl laurate as the acyl donor, acetone as the solvent and lipase B as the catalyst to enable the formation of an ester bond between fructan and lauric acid.

Agave fructans rich in fructooligosaccharides were kindly provided by Nutriagaves (Guadalajara, Mexico). The analysis of agave fructans has already been reported [40]. Vinyl laureate with >99% purity was acquired from Sigma-Aldrich (Burlington, MA, USA), acetone with ≥99.5% purity from Macron-Avantor (Radnor, PA, USA), and immobilized lipase B from Candida antarctica Lipozyme 435™ was obtained from Novozymes (Bagsværd, Denmark). The fructooligosaccharides, vinyl laureate, Lipozyme 435™, and reaction solvent (acetone) were combined, and the mixture was agitated for 48 h at 45 °C. At the end of the reaction, the acetone fraction was filtered, and the enzyme was recovered. Acetone was removed using a Buchi™ rotary evaporator (R-100) (Flawil, Switzerland). Acylation was confirmed in accordance with a previously described process [40]. In brief, reverse high-performance liquid chromatography–diode array detection (HPLC-DAD) was performed using a Luna™ C18 column from Phenomenex (Monterrey, Mexico) in an Acquity Arc HPLC™ system by Waters (Milford, MA, USA). The mobile phase was methanol–water (90:10, *v*/*v*) at a flow rate of 0.5 mL/min. The presence of laurate-bioconjugated fructans was confirmed by the appearance of one peak, and the vinyl laureate peak disappearance.

### 4.2. High-Fat Diet and Food Intake

The high-fat diet (#102238) was acquired from Dyets (Bethlehem, PA, USA). This diet contained 4.6 kcal/g and 45.7% energy from fat. The standard diet (A30) contained 3.4 kcal/g and 14.1% energy from fat and was provided by Safe (Augy, France). Food intake was calculated every day by subtracting the remaining food from the fresh portion served. Food and water were provided ad libitum.

### 4.3. Experimental Animals

Thirty 8-week-old male Wistar rats (219.5 ± 22.9 g) that were exposed to 12 h:12 h light/dark cycles at 22 °C and constant humidity were randomly assigned to five groups (*n* = 6). The initial measurements of one group were designated as time 0 and served as the baseline. The remaining groups were treated as follows. The NORMAL group received a standard diet for 9 weeks, the HFD group was fed a high-fat diet for 9 weeks, the FL PREV group was fed a high-fat diet plus laurate-bioconjugated fructans for 9 weeks, and the FL REV group received a high-fat diet for 6 weeks, followed by simultaneous exposure to the same high-fat diet and laurate-bioconjugated fructans for 3 additional weeks to analyze the reversal effects of laurate-bioconjugated fructans. Laurate-bioconjugated fructans (130 mg/kg) were administered in combination with 3% Tween 80 as a vehicle.

This protocol was approved by the Internal Committee for the Care and Use of Laboratory Animals (CICUAL) (No. 2023-005A) and followed the technical specifications for the production, care, and use of laboratory animals in accordance with the Official Mexican Standard NOM-062-ZOO-1999 [77].

### 4.4. Blood Pressure

At the baseline and after 8 weeks of intervention, tail-cuff plethysmography was performed using a non-invasive blood pressure method with a CODA monitor from Kent Scientific Corporation (Torrington, CT, USA) [40]. The rats were acclimated to the restraint during the week prior to the measurement. The ambient and tail temperatures were controlled. The measurements were recorded five times, and the systolic blood pressure was reported as the mean ± standard deviation.

### 4.5. Zoometric Measurements, Histological Analysis, and Sample Collection

The animals were weighed weekly. The animals were fasted for 4 h and anesthetized with 80 mg/kg i.p. pentobarbital at 0 and 9 weeks of treatment. The length from the nose to the anus was measured at the end of the intervention. The abdominal circumference was also measured; this was reported in cm.

Retro-orbital plexus blood (3 mL) was collected and centrifuged to determine the metabolic and inflammatory serum marker levels. Adipose tissue was obtained before sacrifice for mRNA expression quantification. Liver and adipose tissues were collected and stained using hematoxylin and eosin. The liver specimens were analyzed by a blinded pathologist and the NAFLD activity score (NAS) was determined [21]. The size of the adipocytes was measured using ImageJ, version 1.54 (Bethesda, MD, USA), in accordance with the methods of Parlee [78]. After sacrifice, the liver tissue was weighed and expressed relative to the body weight. Visceral adipose tissue (mesenteric, retroperitoneal, epididymal, and perirenal fat depots) was then collected, weighed, and reported as visceral adipose tissue (g/100 g body weight). Serum and visceral adipose tissue samples were stored at −80 °C.

### 4.6. Biochemical Determinations

Serum glucose, total cholesterol, and triglycerides were determined using colorimetric methods with commercial kits provided by Randox Laboratories (Crumlin, UK). Circulating adipokines and insulin were quantified using an enzyme-linked immunosorbent assay (ELISA), following the manufacturer’s instructions for each kit. The insulin kit was obtained from Millipore (Burlington, MA, USA; Cat. No. RAB0904) and the adiponectin and CRP kits were acquired from ALPCO (Salem, NH, USA; Cat. Nos. 22ADPRT-E01 and 41-CRPRT-E01, respectively). The IL-7 kit was obtained from ABCAM (Waltham, MA, USA; Cat. No. ab277721). The following ELISA kits were acquired from Invitrogen (Carlsbad, CA, USA): IL-6 (Cat. No. BMS625), IL-10 (Cat. No. ERA23RB), and TNF-α (Cat. No. KRC3011). Adipokine measurements were obtained in duplicate using an xMark Microplate Absorbance Reader from Bio-Rad (Hercules, CA, USA).

The determination of insulin resistance was calculated using the homeostasis model assessment of insulin resistance (HOMA-IR) as follows: fasting glucose concentration (mmol/L) × fasting insulin concentration (μUI/mL)/22.5 [17,79].

### 4.7. RNA Isolation and Synthesis of Complementary DNA

Total RNA was isolated from visceral adipose tissue samples with a Pure Link RNA mini kit from Life Technologies (Carlsbad, CA, USA). Briefly, tissue samples were ground and lysed with a hand-held homogenizer in the presence of guanidinium isothiocyanate [80]. After centrifugation, the supernatant was transferred to a cartridge with a silica-based membrane [81], ethanol was added, and the solution was thoroughly mixed. Afterwards, the sample was spun, and the RNA remained bound to the silica. Finally, the RNA was washed three times and eluted using RNase-free water. RNA quantification was performed using a Nanodrop 2000 Thermo Scientific spectrophotometer (Waltham, MA, USA). The RNA was stored at −80 °C until required.

Complementary DNA (cDNA) synthesis was performed using a high-capacity cDNA reverse transcription kit from Applied Biosystems (Foster City, CA, USA). Reverse transcription was ascertained in accordance with the manufacturer’s instructions in a reaction volume of 20 µL. The reaction contained 2 µg RNA, a reverse transcription buffer, MultiScribe™ reverse transcriptase, random primers, an RNase inhibitor, and RNase-free water. This was incubated at 25 °C for 10 min, 37 °C for 120 min, and 85 °C for 5 min. The resulting cDNA was stored at −20 °C until required.

### 4.8. Analysis of Gene Expression

The gene expression was analyzed using a quantitative reverse transcriptase–polymerase chain reaction in a real-time thermocycler (Rotor Gene Q, Qiagen) (Venlo, The Netherlands) with the following TaqMan^®^ probes from Thermo Scientific (Waltham, MA, USA): IL-6 (Rn99999011_m1), TNF-α (Rn00562055_m1), adiponectin (Rn00595250_m1), IL-10 (Rn01644839_m1), IL-7 (Rn00681900_m1), and 18S (4333760T). TaqMan^®^ was selected due to its numerous advantages [82]. The reactions were performed using a total volume of 20 μL, which comprised 10 μL TaqMan^®^ Master Mix, 1 μL of the specific TaqMan^®^ probe, 2 μL cDNA, and 7 μL water. The samples were heated at 50 °C for 2 min and 95 °C for 10 min, followed by 40 cycles at 95 °C for 15 s and 60 °C for 1 min. Gene amplification was normalized against the 18S expression [83]. The samples were analyzed in duplicate. Relative quantification was performed using the comparative relative expression cycle threshold (2^−ΔΔCT^) method. The HFD group was used as an internal calibrator [84].

### 4.9. Statistical Analysis

The data are presented as the means ± standard deviations. The statistical significance was determined for the parametric data using a one-way ANOVA and Tukey’s post hoc test. The Mann–Whitney U test was used for the non-parametric data. A *p*-value < 0.05 was considered to indicate a statistical significance.

## 5. Conclusions

Laurate-bioconjugated fructans could be useful to counteract the adverse effects of a high-fat diet on the characteristics of MetS such as obesity, insulin resistance, high blood pressure, and hypertriglyceridemia, probably by decreasing food intake and modulating CRP and IL-10. Our findings offer additional evidence for the use of laurate-bioconjugated fructans to treat and prevent obesity, obesity comorbidities, insulin resistance, type 2 diabetes mellitus, and MetS.

## 6. Patents

The processes described in patent MX 358,789 were used to synthesize agave fructan bioconjugates.

## Figures and Tables

**Table 1:** Food consumption.

	Baseline	NORMAL	HFD	FL PREV	FL REV
Food intake (g/day)	11.8 ± 2.5	24.6 ± 2.4	18.4 ± 2.1	14.8 ± 2.5 ****	16.5 ± 3.6 ****
Energy intake (kcal/day)	40.0 ± 8.4	83.8 ± 8.2	84.7 ± 9.5	68.2 ± 11.6 **** ^####^	76.0 ± 16.7 **** ^###^
Energy intake from fat (kcal/day)	5.6 ± 1.2	11.8 ± 1.2	38.7 ± 4.3 ^####^	31.2 ± 5.3 ****	34.7 ± 7.6 ****
Energy intake from fat (%kcal)	14.1	14.1	45.7	45.7	45.7

Values are the mean ± standard deviation. Groups: baseline, measurements at week 0; NORMAL, standard diet for 9 weeks; HFD, high-fat diet; FL PREV, high-fat diet for 9 weeks plus laurate-bioconjugated fructans; FL REV, high-fat diet for 6 weeks then simultaneous high-fat diet plus laurate-bioconjugated fructans for 3 additional weeks. *n* = 6 per group. **** *p* < 0.0001 vs. HFD. ^###^ *p* < 0.001 vs. NORMAL. ^####^ *p* < 0.0001 vs. NORMAL. The baseline group was not included in the statistical analysis.

**Table 2:** Metabolic and zoometric parameters.

	Baseline	NORMAL	HFD	FL PREV	FL REV
Glucose (mg/dL)	105.1 ± 14.6	126 ± 22.8	148.3 ± 12.4	134.3 ± 14.6	125.8 ± 22.2
Total cholesterol (mg/dL)	64.2 ± 6.4	57.4 ± 6.5	67.7 ± 7.3	63.9 ± 10.8	70.7 ± 10.6
Triglycerides (mg/dL)	49.8 ± 12.5	68.2 ± 13.4	99.1 ± 15.2 ^#^	66.7 ± 24.9	60 ± 28.8
Adiponectin (μg/mL)	9.8 ± 1.7	6.1 ± 0.8	7.4 ± 0.8	6.9 ± 0.5	7.7 ± 0.9
TNF-α (pg/mL)	9.8 ± 2.4	19.5 ± 7.5	19.1 ± 9.9	19.4 ± 5.5	18.5 ± 3.6
IL-6 (ng/mL)	1.5 ± 0.2	3.2 ± 0.8	4.7 ± 1.7	3.3 ± 0.7	3.9 ± 0.9
IL-7 (ng/mL)	89.7 ± 49.3	175.3 ± 47.9	179.1 ± 88.9	157.9 ± 57.4	290.2 ± 33.6
CRP (μg/mL)	292 ± 30.4	320.3 ± 8.1	335.6 ± 24.2	266 ± 49.5 *	290.2 ± 33.6
Abdominal circumference (cm)	16.8 ± 0.3	19.1 ± 0.3	19.7 ± 1.1	17.8 ± 0.7 **	18.8 ± 0.9
Length (cm)	18.9 ± 0.3	26.3 ± 0.3	27.2 ± 0.4 ^##^	26.4 ± 0.5 *	26.8 ± 0.3
BMI (g/cm^2^)	0.56 ± 0.0	0.67 ± 0.0	0.72 ± 0.0 ^#^	0.60 ± 0.0 ****	0.64 ± 0.0 **

Values are the mean ± standard deviation. Baseline: measurements at week 0. NORMAL: standard diet for 9 weeks. HFD: high-fat diet. FL PREV: high-fat diet for 9 weeks plus laurate-bioconjugated fructans. FL REV: high-fat diet for 6 weeks then simultaneous high-fat diet plus laurate-bioconjugated fructans for 3 additional weeks. *n* = 6 per group. * *p* < 0.05; ** *p* < 0.001; **** *p* < 0.0001 vs. HFD; ^#^ *p* < 0.05; ^##^ *p* < 0.01 vs. NORMAL. The baseline group was not included in the statistical analysis.

**Table 3:** Hepatic histological features.

	Grade	Baseline	NORMAL	HFD	FL PREV	FL REV
Steatosis	0/1/2/3	6/0/0/0	6/0/0/0	2/2/2/0	2/2/2/0	2/2/2/0
Ballooning	0/1/2	6/0/0	6/0/0	0/6/0	1/5/0	0/6/0
Lobular inflammation	0/1/2/3	6/0/0/0	6/0/0/0	6/0/0/0	6/0/0/0	6/0/0/0
NAS score		0 ± 0.0	0 ± 0.0	2 ± 0.9 ^##^	1.8 ± 1.0	1.8 ± 0.4

Liver damage was classified as steatosis (grades 0–3), ballooning (grades 0–2), or lobular inflammation (grades 0–3). The data represent the number of animals in the group at the assigned score. The non-alcoholic fatty liver disease (NAFLD) activity score (NAS) was calculated for each animal and the mean ± SD is presented. ^##^ *p* < 0.01 vs. NORMAL. The baseline group was not included in the statistical analysis.

## Data Availability

Datasets generated or analyzed during the study will be available upon request. The data are not publicly available due to practical limitations.
